# Research progress on the electrophysiological indicators to predict the efficacy of vagus nerve stimulation for drug-refractory epilepsy

**DOI:** 10.1186/s42494-023-00147-y

**Published:** 2024-03-01

**Authors:** Guangli Li, Zhenguang Li, Yingting Liu

**Affiliations:** 1 Hunan University of Chinese Medicine, Changsha, 410208 Hunan Province China; 2 Epilepsy Center of Brain Hospital of Hunan Province, Changsha, 410007 Hunan Province China

**Keywords:** Electrophysiology, Vagus nerve stimulation, Drug-refractory epilepsy, Efficacy, Predictive indicators, Electroencephalogram, Event-related potential, Magnetoencephalography, Aryngeal muscle evoked potential, Heart rate variability

## Abstract

Vagus nerve stimulation (VNS) is an important treatment option for drug-refractory epilepsy (DRE), with well-established efficacy and safety in clinical practice for more than 20 years. However, it is very difficult to find the optimal electrophysiological indicators for the effectiveness of VNS on DRE because the mechanism of action is unknown. In this review, we provide an update of the potential applications of VNS outcomes in patients with drug-resistant epilepsy. Electroencephalographic (EEG) activity, event-related potentials, EEG synchronization levels, magnetoencephalographic, laryngeal muscle evoked potentials, and heart rate variability are potential biomarkers for VNS outcomes in people with DRE.

## Introduction

Epilepsy affects nearly 1% of the world’s population [1]. In addition more than 25–30% epilepsy patients suffer from refractory epilepsy that cannot be controlled by current medications, leading to secondary injuries, illnesses, social dysfunction, and decreased life expectancy. Drug-resistant epilepsy (DRE) is defined by International League Against Epilepsy (ILAE) as the failure to respond to two (or more) tolerated, appropriately chosen, and appropriately used antiseizure medication regimens (whether administered as monotherapies or in combination) to achieve seizure freedom [2]. Due to the lack of a clear epileptogenic focus, patients with DRE are not candidates for surgical resection of epileptic focus after standardized surgical evaluation. It is on this background that one must consider the use of vagus nerve stimulation (VNS). Currently, the efficacy and safety of VNS have been fully validated, but the efficacy of VNS in the treatment of DRE cannot be effectively predicted because its mechanism of action has not been fully understood. With an objective and reliable predictor for the efficacy of VNS,  clinicians can treat DRE patients with a more rational and personalized treatment plan. Neurophysiological indicators are objective and reliable, and can serve as predictors as demonstrated by a large number of studies in recent years.

## Electroencephalogram (EEG) activity and VNS efficacy

EEG is widely used in clinical practice. As a paroxysmal nature of seizure disorders, interictal epileptiform discharges are often used to support the diagnosis of epilepsy and for the preoperative evaluation of refractory epilepsy [3]. Researchers have performed preoperative and postoperative EEG tests in patients with VNS and found that some EEG activities are closely related to VNS outcomes.

### Interictal epileptic discharges (IEDs)

IEDs, also known as subclinical epileptiform discharges, display as a waveform consisting of multiple compound waves and while no obvious clinical symptoms are seen [4]. Janszky et al. followed 47 DRE patients with VNS treatment for at least 1 year and found that 83% of patients with unilateral IEDs achieved seizure freedom. Unilateral IEDs were revealed to be a favorable predictor of seizure freedom by single predictor analysis (sensitivity:83%, specificity:80%) and therefore were considered to be significantly associated with effective VNS treatment [5]. Ghaemi et al. [6] followed 144 patients with epilepsy for at least 2 years after VNS and found that unilateral IEDs were significantly associated with seizure freedom (*P* = 0.005) and significantly predicted seizure freedom post-VNS (*P* = 0.014). This study also showed that multifocal IEDs were negatively associated with VNS treatment outcomes (*P* = 0.007). Kim et al. found that 72.5% of children with focal or multifocal IEDs had a significant treatment effect, whereas only 27.5% of children with generalized IEDs had a significant treatment effect [7]. Multifactorial analysis showed that the VNS treatment outcome is significantly associated with focal or multifocal epileptiform discharges during interictal EEG (*P* = 0.021). Both Marras et al. and Dede et al. reported that focal or multifocal epileptic activities are associated with improved prognosis in epilepsy [8]. In summary, unilateral or non-comprehensive IEDs during preoperative EEG is a favorable factor for VNS outcomes, and patients with focal IEDs may have better VNS outcomes than those with multifocal.

### EEG Power spectrum

The EEG power spectrum expresses the frequency components of the signal or the distribution of signal power over frequency [9]. The power spectral analysis of different frequency components can be used to reflect the EEG responsiveness [10]. Brázdil et al. [11] performed a retrospective power spectral analyses on 60 patients treated with VNS and revealed significant differences between responders and non-responders in two frequency bands (α and γ) and four states (hyperventilation, flash stimulation, eyes open or closed, rest). The activities of α- and γ-waves was significantly increased during hyperventilation in the responders. They further developed a prediction model for VNS efficacy, which showed an accuracy of 86%, sensitivity of 83%, and specificity of 90% in an independent data set of 22 additional patients. The authors concluded that changes in the α- and γ-wave power spectra in the four states could be a potential predictor for assessing patient responsiveness to VNS. The study by Yokoyama et al. [12] investigated whether and how VNS reduces the epileptogenic activity in the bilateral cerebral cortex in patients with intractable epilepsy. They analyzed the electrocorticograms (ECoGs) of five patients after VNS implantation and compared the ECoG background power spectra between the VNS OFF and ON phases. The spectral power in the high-frequency band tended to be greater at the VNS ON phase than the OFF phase and results showed that VNS reduced epileptogenic spikes and the spread areas of the spikes [12].

### Single-channel paired-derived brain symmetry index (pdBSI)

The single-channel pdBSI is one of the measures of EEG symmetry and assesses the symmetry of paired left and right hemispheric homologous channels [13]. de Vos et al. retrospectively analyzed the awake closed-eye EEGs of 19 patients with medically refractory epilepsy over 16 years of age before VNS implantation and calculated the pdBSI in the four frequency bands δ (0.5–4 Hz), θ (4–8 Hz), α (8–12 Hz), and β (12–30Hz) [14]. The results showed that the pdBSI values in all four frequency bands were greater in all patients than in the healthy controls. The pdBSI values of the non-responders were significantly higher than those of the responders. The authors concluded that the patients with lower pdBSI may have better responses to VNS. However, contradictory results have also been reported. In the study by 39 patients with medically intractable epilepsy were classified into good, moderate, and poor categories based on their outcomes after one year of VNS treatment [15]. They retrospectively compared the pdBSI of preoperative EEG in both open- and closed-eye conditions in the three categories, but revealed no significant differences. The authors ultimately concluded that pdBSI is not suitable for predicting the VNS efficacy. Therefore, it remains to be considered whether pdBSI can be used as a potential biomarker for predicting the VNS efficacy.

### Non-rapid eye movement sleep stage 3 (NREM3)

The functional brain organization during sleep exhibits different synchrony and network properties compared to that at wakefulness [16]. Hödl et al. studied the relationship between sleep waves and VNS responsiveness using polysomnography and noted statistically significant differences in preoperative amount of deep sleep (NREM3) between responders and non-responders [17]. Vespa et al. retrospectively analyzed the EEG of 24 epileptic patients treated with VNS (11 responders and 13 non-responders) during calm wakefulness and N3-stage sleep [16].By comparing the weighted phase lag index (PLI), and the global efficiency (GE) between responders and non-responders under VNS ON and VNS OFF conditions, researchers found that the VNS-induced theta wave desynchronization was stronger (*P* < 0.05) and GE was lower (*P* < 0.05) in responders during sleep, while no significant changes were found during wakefulness [16, 18]. However, more studies are needed to verify these results and confirm whether sleep waves can be used to predict the efficacy of VNS. 

## EEG synchronization level and VNS efficacy

Seizures are closely associated with neuronal hypersynchronous neuronal discharges. Desynchronization of neuronal discharges is a key mechanism underlying the efficacy of VNS in epilepsy, and EEG synchronization measurements have been explored as possible predictors of response ro VNS [19].

### Phase lag index (PLI)

Fraschini et al. retrospectively compared the preoperative synchronization levels of interictal EEG activity in different brain regions between VNS responders and non-responders (32–57 years old) using the PLI method [20], which is not affected by homozygosity, and found no statistically significant difference in the whole-brain mean PLI in different frequency bands between the two groups. Bodin et al. also evaluated the level of synchronization of interictal EEG activity in 19 patients (14–54 years old) after VNS using the PLI method and found the PLI values in the δ and α frequency bands were lower in those who responded to VNS compared to those who did not respond (*P* = 0.02) [21], suggesting that PLI may be a potential indicator to predict the prognosis of VNS treatment.

### Cortical synchronization index (SI)

The synchronizability of each EEG electrode is defined as the contribution of each electrode to the global network synchronization. The number of electrodes that achieve a statistically significant increase in the SI during seizures can be used as a feature to study the effects of VNS on EEG spatial synchronization. Ravan et al. used the SI of electrodes to assess the level of synchronization of EEG activity during the interictal period in 15 patients (18–69 years) [22]. They found that the SI of all electrodes before VNS had a significant increase in the interictal period compared to baseline, while after VNS, in some patients, the SI of electrodes at different locations was still significantly increased in the interictal period compared to baseline, suggesting that the locations at which VNS reduces the synchronization level in brain regions during the interictal period vary among individual patients. In a follow-up by the same authors, the percentage of electrodes with a significantly different increase in SI for a single postoperative seizure to the total number of electrodes showed a 93.33% accuracy in predicting the long-term efficacy of VNS in patients (21–50 years) at 1 year after operation based on EEG collected over 1 month after VNS [23].

The above study suggests that the PLI may only be used for prognostic assessment of surgery and that the SI does not change much after VNS, suggesting that it may not be a good predictor of the efficacy of VNS for DRE. Overall, patients with higher levels of desynchronization after VNS had better long-term outcomes.

## Event-related potential and VNS efficacy

### P300

P300 is a positive component with a latency of approximately 300 ms that can be observed in the EEG under specific stimulations and can reflect temporal changes in the neuromodulation of norepinephrine [24]. deTaeye et al. designed an oddball stimulation sequences to observe changes in the P300 component in 20 patients (21–66 years) and found that in the VNS responsive group, the postoperative P300 wave amplitude of the parietal midline electrode was significantly higher than the non-responsive group (*P* = 0.017), and the difference in the wave amplitude between the ON and the OFF states was significant in the responsive group (*P* = 0.007) [25]. Logistic regression analysis suggested that the changes in P300 wave amplitude are a better predictor. Wostyn et al. further found that the P300 wave amplitude change in the CP2 lead in the OFF state combined with that of the PO5 lead in the ON state had an accuracy of 94%, which was higher than the accuracy of the P300 wave amplitude change in a single parietal midline lead (61%). They also observed an increase in P300 amplitude in  the VNS responders only [26].

### Slow cortical potentials (SCPs)

SCPs are a specific ERP component which indicates inhibition of postsynaptic potentials and neuronal activity, and their generation is associated with epileptic inhibition. Bayasgalan et al. analyzed the conventional clinical EEG of 24 epileptic patients treated with VNS [27].According to the EEG hardware time constant (TC), the correlation between SCPs (positive or not) and seizure reduction (> 50% or not) was estimated using seizure reduction as an independent judgment indicator. They found that in the TC10-s group, the correlation between SCPs and seizure reduction was significant (*P* < 0.05); while in the TC2-s group, the correlation was not significant (*P* = 0.209), verifying that a positive shift of SCPs can be a marker for response to VNS.

In summary, P300 and SCPs have shown potentials to predict the efficacy of VNS for DRE. Moreover, the P300 wave amplitude changes are significantly different between the preoperative and the postoperative periods in VNS-responders, suggesting that they may serve as a better predictors.

A further search of databases such as PubMed and CNKI, for studies on the use of EEG indicators to predict the VNS efficacy resulted in a small number of literature published either in English or in Chinese language (Table 1).

**Table 1:** Number of studies searched in PubMed and CNKI on the use of EEG indicators to predict the VNS efficacy

Database	Keywords	Number of studies	Summary
PubMed	"EEG" and "VNS"	28	Studies were focused on the role of EEG in the prediction of VNS efficacy
	"EEG", "VNS" and "predictive"	11	Clarifying the role of EEG in the prediction of VNS efficacy
CNKI	"EEG" and "VNS"	30	Studies were focused on the use and efficacy of EEG and VNS in the treatment of epilepsy,
	"EEG", "VNS" and "predictive"	4	Four papers explored the use of EEG in the prediction of VNS, three of which clarified the ability of EEG to predict the efficacy of VNS

## Magnetoencephalography (MEG) and VNS efficacy

### Resting-state magnetoencephalography (rs-MEG) connectivity analysis

rs-MEG connectivity analysis has been increasingly used to study the effects of epilepsy on brain networks and to identify changes in these networks after different treatments. Babajani-Feremi et al. investigated whether the rs-MEG network topology prior to VNS implantation could be used to predict the efficacy of VNS treatment [28]. Their study included 23 epileptic patients who received MEG prior to VNS implantation, using phase-locked values in the θ, α and β bands as a measure of rs-MEG functional connectivity. Three global graph metrics were also calculated: modularity, transmissibility, and characteristic path length (CPL). The results showed that the rs-MEG graph measures were significantly transferable and had an overall good retest reliability. They also found that the modularity was greater in the VNS responders than in the non-responders, while transmissibility was smaller in the VNS responders than in the VNS non-responders. In addition, the modularity and transmissibility in the three frequency bands and CPL in the δ and β bands were significantly different in healthy controls than in the VNS responders or VNS non-responders, and that graph measures in controls were closer to those of the VNS responders. The study by Wang et al. included 20 pharmaco-resistant Dravet syndrome (DS) patients to receive VNS implantation and they were classified into responder and non-responder groups at 24 months post-VNS. Brain parameters (CPL,GE and transitivity) in two frequency categories (α and β) on rs-MEG between 6 months pre- and 6, 12, and 24 months post-VNS were analyzed in all patients, responders, and non-responders. The results showed that during the long-term follow-up, the responders had a decreased transitivity after the VNS treatment and that the difference in the transitivity between responders and non-responders is more pronounced than the differences in CPL and GE in both α (*P* < 0.015) and β (*P* < 0.001) bands [29]. In the study by Mithani et al. 56 children were included into discovery (*n* = 38) and validation (*n* = 18) cohorts. Diffusion tensor imaging was used to identify differences between responders and non-responders in white matter microstructure, which in turn informed beamforming of rs-MEG recordings. The resulting classifier demonstrated 89.5% accuracy and an area under the receiver operating characteristic curve (ROC) of 0.93 on 10-fold cross-validation. In the external validation cohort, this model demonstrated an accuracy of 83.3%, with a sensitivity of 85.7% and specificity of 75.0%. This was significantly superior to predictions using clinical covariates alone [30]. Thus, MEG-based graph measurements are reliable biomarkers to predict seizure outcomes with VNS treatment.

### Somatosensory evoked fields (SEFs)

A recent study by Mithani et al. evaluated the use of SEFs generated by median nerve stimulation during MEG recordings to predict response to VNS [31]. The study adopted retrospective data from 48 children treated with VNS at two different institutions. Thirty-six patients ("discovery cohort") received preoperative median nerve electrical stimulation during MEG recordings, and 12 patients ("validation cohort") received preoperative pneumatic stimulation during MEG. SEFs and their spatial deviations, waveform amplitudes and latencies, and event-related connectivity were calculated for all patients. Results showed that more widespread SEFs localization correlated with the responsiveness to VNS, while the amplitude latency of SEFs did not. In addition, there were significant event-related functional connectivity differences within limbic and sensorimotor networks in the VNS responders, compared to the non-responders. Finally, after leveraging overlapping neural circuitry, the median nerve SEFs features and functional connectivity identified responders to VNS.

## Laryngeal muscle evoked potentials (LMEPs) and VNS efficacy

Non-invasive evoked potential recordings (LMEPs) of the vagally innervated laryngeal muscles may provide a marker measure to assess effective vagal nerve fiber activation. Bouckaert et al. studied VNS-induced LMEPs in patients with acute and chronic epilepsy [32]. The VNS-induced LMEPs were recorded according to different pulse widths and output currents using six surface laryngeal muscle electrodes. Input/output curves were calculated and the latency of LMEPs was estimated. The threshold current for minimal, half-maximal, and 95% of maximal response induction and the amplitude of maximal response (V_max_) were compared with VNS responders and VNS non-responders in the acute and chronic groups. The VNS-induced LMEPs were observed in all patients, and there was no significant difference between VNS responders and VNS non-responders. However, V_max_ was lower in all patients after one year compared to baseline. Therefore, noninvasive recording of VNS-induced LMEPs at the start of VNS treatment and after one year may be a predictor of the efficacy of VNS for DRE.

## Heart rate variability (HRV) and VNS efficacy

Current studies have shown that epilepsy onset and progression is accompanied by impairment of the autonomic nervous system, which can lead to a decrease in HRV [33]. In a retrospective analysis of HRV-related indicators before and after VNS in 32 patients with DRE (6–38 years), Liu et al. found that the preoperative HRV indicators in responders did not differ from healthy controls [34], while those in non-responders (except for the mean RR interval and low-frequency to high-frequency energy ratio) were significantly lower compared to healthy controls. Further analysis of preoperative HRV measurements in an additional 63 patients with DRE (5–38 years) and ROC curve analysis revealed that in patients 6—20 years of age, short-term variability caused by respiration and the square root of the mean of the sum of the squares of the differences between adjacent RR intervals are good predictors of seizure reduction after VNS and patients with > 50% reduction in seizures from baseline [34]. In a second study [35], the authors repeated these results in the same group. They also demonstrated lower preoperative frequency domain measures in VNS non-responders and a lower preoperative complexity index in VNS responders compared to VNS non-responders. Hödl et al. reported lower HF power in responders compared to VNS non-responders before VNS treatment and 1 year after VNS treatment. In conclusion, patients with higher preoperative HRV-related indexes are more likely to benefit from VNS [36].

In summary, patients with higher preoperative HRV-related indexes are more likely to benefit from VNS.

## Conclusion and outlook

VNS, as a palliative procedure, largely reduces seizure frequency and shortens seizure duration in patients with DRE and  those who cannot be craniotomized. It has also been shown to substantially (> 30%) reduce the risk of sudden unexpected death in epilepsy [37], improve cognitive function, and improve the quality of life of patients in terms of mood regulation. As it is relatively expensive and may be completely ineffective in approximately 10% of patients [38], it is important to preoperatively determine whether patients with DRE will benefit from VNS. Electrophysiological indicators have been recommended to be used to predict the efficacy of VNS. For example, EEG activity (IEDs power spectrum, single-channel pdBSI, sleep waves, ERPs-P300, SCPs, EEG synchronization levels (PLI, cortical SI), MEG, SEFs, LMEPs, and HRV may be used for the prediction of VNS efficacy and may also be a long-term prognostic indicator for improved episodes of clinical DRE. More importantly, patients with EEG activities such as preoperative unilateral or focal interictal epileptiform discharges, higher levels of postoperative desynchronization, greater structural and functional connectivity between brain regions, and higher HRV-related indicators would benefit more from VNS as a treatment. The postoperative EEG synchronization level, the wave amplitude of P300 and the LMEPs can be used as biomarkers to predict the long-term prognosis of VNS.

Although existing studies have yielded many results, they still have certain limitations. On the one hand, the limited number of patients with VNS and the lack of relevant literature have led to the inability to clearly determine the validity of neurophysiological indices, and more in-depth studies are urgently needed. On the other hand, most of the studies were retrospective single-center studies, and the results are influenced by factors such as the times of VNS treatment and the participation of patients in independent validation, thus compromising the stability and maturity of the obtained indicators; therefore, the research results at this stage are inadequate and must be validated in prospective, multicenter and well-designed long-term clinical studies.

## Data Availability

Not applicable.

## Abbreviations

VNS: Vagus nerve stimulation
DRE: Drug-refractory epilepsy
EEG: Electroencephalographic
ERPs: Event-related potentials
MEG: Magnetoencephalographic
LMEPs: Laryngeal muscle evoked potentials
HRV: Heart rate variability
IEDs: Interictal epileptic discharges
ECoGs: Electrocorticograms
pdBSI: Paired-derived brain symmetry index
NREM3: Non-rapid eye movement sleep stage 3
PLI: Phase lag index
SI: Synchronization Index
EPR: Event-related potential
P300: A positive component with a latency of approximately 300 ms
SCPs: Slow cortical potentials
rs-MEG: Resting-state magnetoencephalography
CPL: Characteristic path length
DS: Dravet syndrome
ROC: Receiver operating characteristic
SEFs: Somatosensory evoked fields
